# Percutaneous radiofrequency ablation with contrast-enhanced ultrasonography for solitary and sporadic renal cell carcinoma in patients with autosomal dominant polycystic kidney disease

**DOI:** 10.1186/s12957-016-0916-3

**Published:** 2016-07-26

**Authors:** Linfeng Xu, Yang Rong, Wei Wang, Huibo Lian, Weidong Gan, Xiang Yan, Xiaogong Li, Hongqian Guo

**Affiliations:** School of Medicine, The Affiliated Drum Tower Hospital of Nanjing University, 321 Zhongshan Road, Nanjing, Jiangsu People’s Republic of China

**Keywords:** Autosomal dominant, Contrast-enhanced ultrasonography, Polycystic kidney, Radiofrequency ablation, Renal cell carcinoma

## Abstract

**Background:**

The aim of this study was to assess the functional and oncologic outcomes of percutaneous radiofrequency ablation (RFA) with contrast-enhanced ultrasonography (CEUS) for renal cell carcinoma in patient with autosomal dominant polycystic kidney.

**Methods:**

We performed a retrospective review of five patients with renal cell carcinoma (RCC) in autosomal dominant polycystic kidney disease (ADPKD) from January 2009 to December 2014 with a media follow-up of 33 months. The tumors were ablated with Cool-tip RFA system under the guidance of CEUS. Routine follow-up included contrast-enhanced computed tomography/magnetic resonance imaging (CT/MRI) and renal function tests.

**Results:**

Media diameter of the treated renal tumors was 3.1 cm (range 1.7–5.2 cm). Initial ablation success rate was 4/5. After over 6 months contrast-enhanced CT/MRI follow-up after RFA, no patients experienced local tumor recurrence. No patients required dialysis in the periprocedural period. Minor complications only developed in two cases. There was no significant difference in estimated glomerular filtration rate (eGFR) between pre- and post-RFA.

**Conclusions:**

Our initial experience of this technique for RCC in ADPKD was favorable with good renal function preservation and oncologic outcomes. It may be a good choice for RCC in ADPKD.

## Background

Autosomal dominant polycystic kidney disease (ADPKD) is a common hereditary renal cystic disease with an incidence of approximately 1:1000 [1], which is mainly caused by mutations of the PKD1 or PKD2 genes [2, 3]. In half of ADPKD patients, the development and growth of cysts could result in early renal functional abnormalities [4]. Most patients died of renal failure and infections [5].

Renal cell carcinoma (RCC) in ADPKD is rare and largely populated by single case reports [6–10]. RCC may occur in ADPKD with normal function or end-stage renal disease (ESRD). It may be located in the unilateral or bilateral kidneys [10]. The most popular pathological types of RCC in ADPKD are clear cell carcinoma or papillary carcinoma [11, 12]. However, the role of ADPKD as a risk factor for renal cell carcinoma (RCC) is still controversial. Whether or not this is due to chronic dialysis or due to the underlying disease is still speculative. The frequency of RCC in ESRD with ADPKD would be two to three times more than that in ESRD alone [12].

So far, the standard treatment of RCC is surgical excision. Radical nephrectomy (RN) and partial nephrectomy (PN) are alternatives with equivalent long-term oncologic and renal functional outcomes. Because of the structural distortion in ADPKD, PN is difficultly performed and RN is the preferable choice. However, ADPKD patients had a high risk of postoperative renal insufficiency if undergoing renal resection. Therefore, a mini-invasive surgical procedure is needed to decrease the loss of renal unit when we confront RCC in ADPKD.

Radiofrequency ablation (RFA) is an evolving nephron-sparing surgery for small renal tumors. RFA does not require even transient clamping of renal vessels and shows the following advantages: less perioperative morbidity, maximal nephron conservation, shorter hospitalization, and convalescence. The current American Urological Association guidelines have recommended RFA as an option for patients with T1 disease. RFA seems to be a good choice for solitary RCC in ADPKD patients.

Herein, we perform a retrospective review of five ADPKD patients with RCC undergoing percutaneous RFA under the guidance of contrast-enhanced ultrasonography from January 2009 to December 2014 with a media follow-up of 33 months.

## Methods

### Patients

**Fig. 1:**
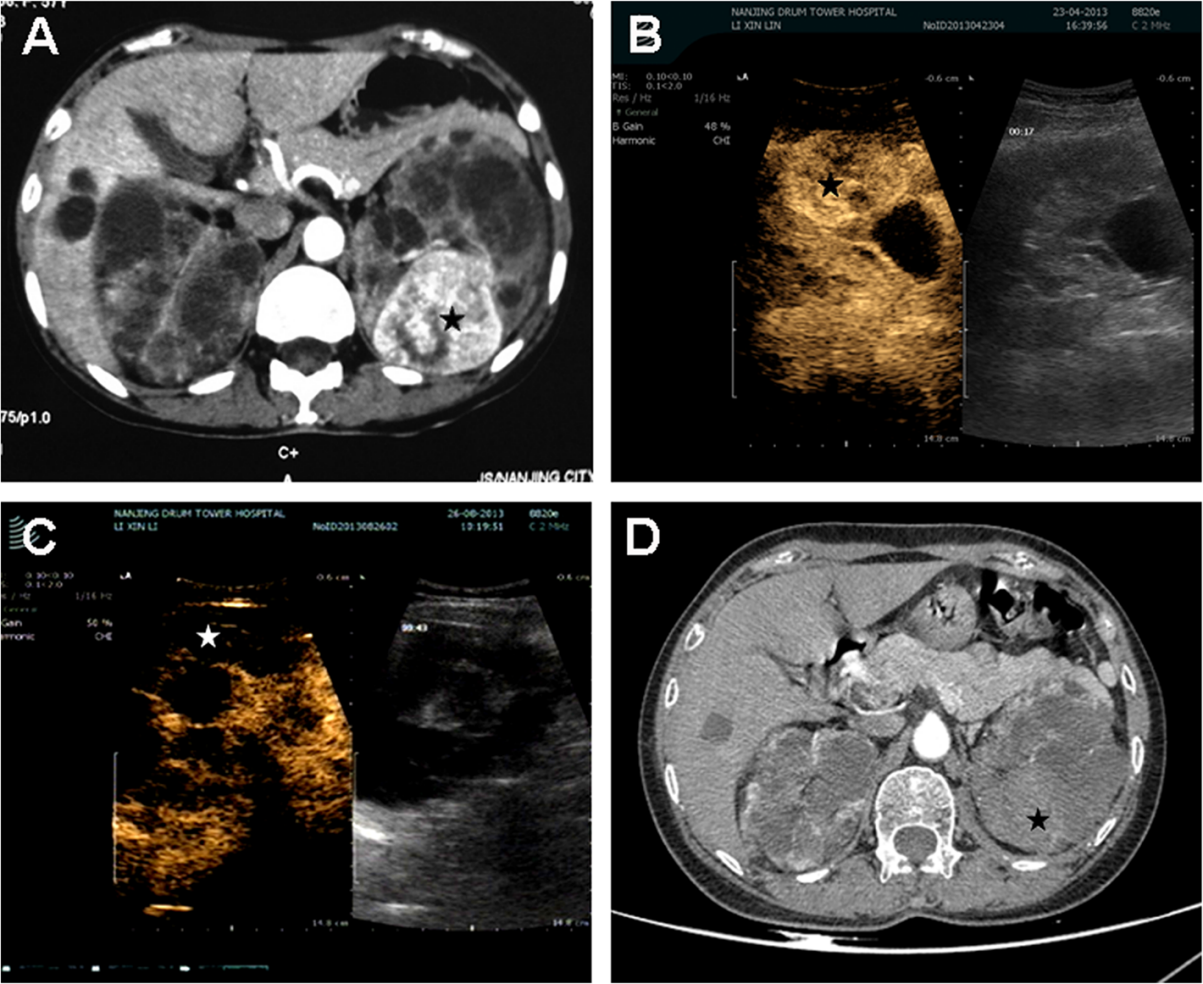
Percutaneous RFA was applied on a 57-year-old female ADPKD patient. The tumor was 5.2 cm in diameter and endophytically located in the posterior medial part of the left kidney. The tumor was completely ablated after two sessions of PRFA. **a** contrast-enhanced CT (arterial phase) obtained before PRFA showed enhancement of the tumor (*black star*). **b** CEUS shows the outline of the tumor (*black star*) before RFA. **c** CEUS was applied right after the final session of ablation and absence of enhancement was found in the tumor’s location (*black star*). **d** contrast-enhanced CT (arterial phase) obtained 1 year after final session of ablation shows absence of enhancement, indicating complete tumor necrosis (*black star*)

From January 2009 to December 2014, ultrasound-guided percutaneous RFA was performed on five ADPKD patients with RCC in two men and three women (age range 55–73 years, media age 57 years) by a single surgeon (Fig. 1). Our research was approved by the Nanjing Drum Tower Hospital’s ethics committee (No. 20090016). Informed consent was obtained from each patient after the surgeon reviewed the peer-reviewed data on cancer-specific survival, recurrence risk, complication rates, and expected convalescence period.

All the tumors were sporadic and solitary. The diameters of the tumors were all <7 cm (range from 1.7 to 5.2 cm). Three cases located in the left kidney. Two cases located at the ventral and upper kidney, one case located at the dorsal and upper kidney, and the rest two located at the dorsal and lower kidney. All tumors were over 1 cm away from renal hilar vessels. The metastatic diseases were evaluated by contrast-enhanced CT and bone scan. All patients were at cT1N0M0 stage with poor renal function and did not want to take the risk of partial nephrectomy (PN). All patients were followed up over 6 months by radiography. The media radiographic follow-up duration was 33 months (range 7–70 months).

### Percutaneous RFA

Patients received general anesthesia and were placed in prone or lateral decubitus position depending on the size and location of the tumors. Contrast-enhanced ultrasonography (CEUS) was performed with ultrasonic device (B/K, Denmark) before RFA to reveal the exact location and outline of the tumor and to define the exact skin location for the electrode insertion. Ultrasonic device was used to guide the initial insertion of the electrode to the deepest margin of treatment. For tumor <3 cm, single electrode (17G) with a maximal ablation diameter of 2 or 3 cm was used; for tumor ≥3 cm, two or more electrodes were simultaneously applied to cover the targeting area. After biopsy (TruCore, 22G), the Cool-tip system (Radionics, Burlington, MA) was applied for RFA. The radiofrequency generator (Radionics) monitored tissue impedance and automatically adjusted the output of maximum energy delivery. The electrode was kept at 15–20 °C by means of internal cooling with chilled water delivered from a peristaltic pump. RFA was performed for a cycle of 12 min per cycle according to the manufacturer’s recommendations. Then, CEUS was applied to evaluate the effect of the previous ablation. Extra cycles were applied if there was highly suspicious residue in the original tumor location. The electrode track was ablated before it was withdrawn.

### Contrast-enhanced ultrasonography

A dose of 1.2–2.4 mL of sulfur hexafluoride in the form of microbubbles (SonoVue, Bracco, Milan, Italy) was injected intravenously, via an antecubital vein, by using a 20-gauge cannula as a rapid bolus followed by a 5-mL saline flush. Harmonic microbubble-specific imaging with low acoustic US pressure (2–4 MHz transducer; mechanical index <0.2; 12–13 frame rate/s) was performed utilizing the same machine. CEUS was performed right after the initial ablation. Additional ablation and CEUS were performed focused on the highly suspicious area.

### Follow-up

There are various follow-up protocols for radiofrequency-ablated renal tumors. Contrast-enhanced CT currently appears to be the most effective method. At our institution, initial evaluation started by CEUS 7 days after the procedure. If residual was found, another session of RFA will be performed 1 month later. Subsequent CT and CEUS assessment were performed at 3 and 6 months postoperatively, and every 6 months thereafter. Patients with renal insufficiency or contrast agent allergy were followed up with gadolinium-enhanced MRI. Successful ablation was conformed when the lesion showed less than 10 HU of contrast medium enhancement on CT or no qualitative evidence of enhancement after intravenous gadolinium contrast-enhanced MRI. Recurrence was defined as any new enhancement (>10 HU) on an enhanced CT. Each patient underwent serum creatinine measurement with every CT examination, and the glomerular filtration rate (GFR) was calculated using the modified modification of diet in renal disease (MDRD) equation: eGFR = 175 × (serum creatinine) − 1.154 × (age) − 0.203 × (0.742 if female) × (1.210 if black).

## Results and discussion

The oncologic and functional outcomes were shown in Table 1. One week after initial RFA, four tumors were considered completely ablated and one tumor of 5.2 cm was considered incompletely ablated through CEUS. The patient with residue underwent the second session of PRFA 1 month later, and the residue was completely ablated proven by follow-up CT scan 3 and 6 months after initial procedure. No patients died, and no recurrence or metastatic diseases were detected during the follow-up period. No serious complications were found in all patients though small volume hematuria (in one patient) and limited perinephric hematoma (in one patient) were found. The pre- and post-RFA mean GFR levels were 54.5 ± 5.4 mL/min/1.73 m^2^ and 53.8 ± 6.0 mL/min/1.73 m^2^ (*P* > 0.05, Student’s *t* test).

**Table 1:** Oncologic and renal function outcomes of patients

Case	Age	Gender	Tumor size (cm)	Complication	Initial classification	Session	Pre-RFA GFR (mL/min/1.73 m^2^)	6 months post-RFAGFR (mL/min/1.73 m^2^)	Follow-up time (m)	Outcome
1	55	M	2.8	Limited perinephric hematoma	Papillary RCC	1	58.9	57.3	70	Free
2	57	F	5.2	None	Papillary RCC	2	53.4	52.2	7	Free
3	55	M	3.2	Small volume hematuria	Papillary RCC	1	61.2	62.3	33	Free
4	63	F	1.7	None	Clear cell	1	48.4	47.2	54	Free
5	73	F	3.1	None	Clear cell	1	50.7	50.1	21	Free

ADPKD is the most common hereditary kidney disease. Patients with ADPKD often present with a lot of cysts in the kidney and decreasing renal function. The prevalence of ADPKD is 1/1000. 9.8 % of patients finally need renal replacement therapy (dialysis or kidney transplantation) in Europe [13].

Although the prevalence is rare, RCC is the most serious complication of ADPKD. The exact relation of ADPKD and RCC has never been confirmed. The prevalence of RCC in ADPKD is surprisingly high. Hajj et al. reported that 11 of 89 kidneys in ADPKD patients were diagnosed with renal cell carcinomas [11]. Jilg et al. reported 12/240 (5 %) patients with ADPKD presented with malignant renal lesions [12]. According to previous literatures, the most popular histologic types in ADPKD patients are papillary RCC and clear cell RCC. There was a higher frequency of the papillary RCC subtype in ADPKD compared with the general population [14].

Because of poor renal function and anatomical problem, surgical therapy for RCC in ADPKD is very tough. No consensus has been established about how to treat it. Radical nephrectomy or nephron-sparing surgery was alternative options for T1 stage RCC at present. However, these two procedures both have inherent defects in ADPKD patients: lots of functional nephrons are lost in radical nephrectomy and unclear tumor boundary and inexact suture of renal tissue limit the use of nephron-sparing surgery due to the distortion of the renal anatomy. Therefore, these two methods are not ideal.

RFA is a kind of technique that uses alternating radiofrequency current to generate localized heat and induce tissue necrosis. It is increasingly applied in the management of small renal tumors with excellent long-term results comparable to nephron sparing nephrectomy. RFA is an effective treatment option for patients with RCCs less than 4 cm who are poor surgical candidates (patients with serious co-morbidities, borderline renal function, solitary kidney, and high recurrence risk [Von Hippel-Lindau disease]) [15]. RFA can be applied by various means: open, laparoscopic, and percutaneous approaches. Percutaneous RFA has been preferred by most urologists and radiologists with minimal invasiveness, short hospitalization, and convalescence period [15–19]. Although the tumor control rate of percutaneous RFA is relatively lower compared with laparoscopic RFA [20], the repeatability could make up the deficiency. Furthermore, it has little influence on renal function and could preserve renal function as much as possible. Sung et al. reported RFA was superior to OPN with respect to the preservation of renal function for treating size- and location-matched RCCs [17]. It seems to be a good option for RCC in ADPKD.

Due to the deformity of renal structure in ADPKD, the extent of renal tumor could be hardly defined under conventional ultrasound. Recently, the development of imaging techniques and new contrast media has leaded to the occurrence of contrast-enhanced ultrasound (CEUS), which has already been used in renal tumor diagnosis and showed obvious superiority over conventional ultrasound (US). CEUS was found to possibly improve the diagnostic confidence of RCC, providing abundant information on tumor vasculature and the blood supply. CEUS was superior to conventional US and CECT in visualizing the number of septa and wall thickness, and the presence of solid component in cystic renal lesions. In addition, the ultrasound contrast agent is relatively harmless with a lower incidence of side effects such as nephrotoxicity. Contrast-enhanced ultrasound has been reported to be useful in depicting residual lesions during and after ablative therapies in various tumors [21, 22]. Zhao et al. reported that the success rate after initial RFA for small renal mass in the CEUS group treatment is significantly higher than that in the conventional ultrasound group (94.6 vs. 84.1 %, *P* < 0.05) [18]. CEUS was first reported to be effective during the follow-up of renal tumor cryoablation and could detect recurrences after cryoablation. Recently, several retrospective studies confirmed that CEUS was an effective alternative to CT and MRI in the follow-up of renal tumors treated with RFA, with some advantages: low cost, short time-consuming procedure, no radiation exposure, reduced amount of contrast agent (1–2 mL), and rare adverse reactions [22, 23].

The ultimate goal of RFA is to completely damage the entire tumor and to preserve renal function as much as possible. As we all know, most solitary and sporadic renal tumors in ADPKD patients were surrounded by renal cysts. The cysts around the tumor would help to prevent thermal damage to the adjacent renal parenchyma and preserve more renal function, which was a big advantage of ADPKD patients treated with RFA. CEUS could further help to define the border of renal tumor and guide the electrode probe placement. Moreover, for highly suspicious residues, CEUS guided complementary RFA targeting on the specific area would avoid damaging the normal renal tissue through modified output of energy delivery. However, these surrounding cysts could also result in “heat sink” effect which would reduce the effectiveness of the ablative procedures. Therefore, it would take more time to perform RFA on renal tumors of ADPKD patients than those of common patients. Meanwhile, the success rate after initial RFA decreased with the increase of tumor sizes. CEUS was definitely important to determine whether the tumor was completely ablated under this circumstance. Second-session RFA should be engaged in patients with large tumor to reduce complications from long time ablation in one session. In our present series, we have successfully ablated small solitary solid renal tumors for one session and tumor >5 cm was completely ablated through two sessions.

Although our patients had a favorable clinical course, there were various limitations in the study. First, the rare and sporadic cases restricted the multi-sample research. It is hard to set a threshold (dimensions or focality) for this intervention with limited cases. Second, we do not have pathologic studies that confirm that the ablations were complete, and imaging techniques have limitations in identifying incomplete ablation and recurrence.

## Conclusions

Our initial experience of this technique for renal cell carcinoma in autosomal dominant polycystic kidney was favorable with good renal function preservation and oncologic outcomes. It may be a good alternative to partial nephrectomy for selected renal cell carcinoma in autosomal dominant polycystic kidney.

## Abbreviations

ADPKD, autosomal dominant polycystic kidney disease; CEUS, contrast-enhanced ultrasonography; CT, computed tomography; ESRD, end-stage renal disease; GFR, glomerular filtration rate; PN, partial nephrectomy; RCC, renal cell carcinoma; RFA, radiofrequency ablation; RN, radical nephrectomy
